# A Machine Learning-Based Model for Preoperative Assessment and Malignancy Prediction in Patients with Atypia of Undetermined Significance Thyroid Nodules

**DOI:** 10.3390/jcm13247769

**Published:** 2024-12-19

**Authors:** Gilseong Moon, Jae Hyun Park, Taesic Lee, Jong Ho Yoon

**Affiliations:** 1 Division of Thyroid-Endocrine Surgery, Department of Surgery, Wonju Severance Christian Hospital, Yonsei University Wonju College of Medicine, Wonju 26426, Republic of Korea; gsmoon@yonsei.ac.kr (G.M.); jhoney@yonsei.ac.kr (J.H.P.); 2 Division of Data Mining and Computational Biology, Institute of Global Health Care and Development, Wonju Severance Christian Hospital, Yonsei University Wonju College of Medicine, Wonju 26426, Republic of Korea; ddasic123@yonsei.ac.kr

**Keywords:** atypia of undetermined significance (AUS), neutrophil-to-lymphocyte ratio (NLR), delta neutrophil index (DNI), platelet-to-lymphocyte ratio (PLR), thyroid cancer, malignancy prediction

## Abstract

**Objectives:** The aim of this study was to investigate the preoperative clinical and hematologic variables, including the neutrophil-to-lymphocyte ratio (NLR), that can be used to predict malignancy in patients with atypia of undetermined significance (AUS) thyroid nodules; we further aimed to develop a machine learning-based prediction model. **Methods:** We enrolled 280 patients who underwent surgery for AUS nodules at the Wonju Severance Christian Hospital between 2018 and 2022. A logistic regression-based model was trained and tested using cross-validation, with the performance evaluated using metrics such as the area under the receiver operating characteristic curve (AUROC). **Results:** Among the 280 patients, 116 (41.4%) were confirmed to have thyroid malignancies. Independent predictors of malignancy included age, tumor size, and the Korean Thyroid Imaging Reporting and Data System (K-TIRADS) classification, particularly in patients under 55 years of age. The addition of NLR to these predictors significantly improved the malignancy prediction accuracy in this subgroup. **Conclusions:** Incorporating NLR into preoperative assessments provides a cost-effective, accessible tool for refining surgical decision making in younger patients with AUS nodules.

## 1. Introduction

Worldwide, fine-needle aspiration (FNA) is the method most commonly and widely utilized to determine the malignancy of thyroid cancer. Its simplicity, safety, cost-effectiveness, and diagnostic accuracy make it the preferred choice. However, FNA has its limitations, with inconclusive results such as non-diagnostic nodules, or the interpretation of nodules as indeterminate nodules, such as atypia of undetermined significance (AUS), follicular neoplasm, or nodules that are suspicious for malignancy.

Among these diseases, AUS is reported to have a 13–30% probability of ultimately being diagnosed as cancer, although there is considerable variability across institutions [1]. This ongoing issue has prompted concerns, with some studies reporting a higher frequency of reclassifications of indeterminate nodules than the reported range. To address these challenges and provide accurate guidance for treatment decisions, various guidelines, including the ATA guidelines, generally recommend repetitive FNA and molecular testing. However, repeated FNA in AUS patients can lead to significant problems, with the literature indicating that more than 60% of results are inconclusive (either reclassified as category I or again as category III). Additionally, despite ongoing research on molecular testing, there is currently no widely used screening tool globally, except for the BRAFV600E mutation, which is used to distinguish papillary thyroid cancer (PTC). In the effort to overcome these challenges, products such as Thyroseq, a pioneering tool for NGS-based multi-gene analysis, are under continuous investigation, but there remain barriers to their widespread use, such as economic constraints.

The most definitive diagnostic approach ultimately involves performing a diagnostic lobectomy. However, the feasibility of surgery itself may be limited based on the patient’s general condition, underlying conditions, and socio-economic background, among other factors. Artificial intelligence (AI) has revolutionized healthcare by enabling systems to process and analyze vast datasets with unprecedented speed and accuracy. In oncology, AI-based tools have demonstrated high specificity and sensitivity, especially in medical imaging analysis, aiding clinicians in identifying malignancies early and accurately [2]. For example, recent studies have demonstrated the use of AI and machine learning in enhancing cancer diagnosis, particularly in colon cancer, by means of bioinformatics analysis and imaging-based approaches [3]. These technologies promise to bridge the existing diagnostic gaps by integrating diverse data points into cohesive decision-making frameworks. Despite these advancements, significant challenges remain in translating AI systems from experimental research to routine clinical practice. Key barriers include concerns around data privacy, the lack of interoperability between healthcare systems, and insufficient validation across diverse patient populations. Addressing these limitations is critical to unlocking the full potential of AI in healthcare.

While AI-based systems offer advanced diagnostic capabilities, simpler and more accessible tools such as hematologic indices have recently gained traction, particularly in resource-limited settings. Hematologic indices such as the delta neutrophil index (DNI), the neutrophil-to-lymphocyte ratio (NLR), and the platelet-to-lymphocyte ratio (PLR) have been validated as predictive markers in thyroid and other cancers, offering cost-effective solutions for preoperative assessments [4,5,6]. Among these, the NLR has shown potential as a marker for predicting differential thyroid cancer [7]. This study explores the potential of using hematologic indices as predictive tools for malignancy in AUS cases. These indices, being both cost-effective and widely accessible, present a promising alternative to advanced molecular testing, particularly in settings where resources are constrained. By focusing on accessible preoperative data, this study aims to address gaps in current diagnostic approaches and support more informed surgical decision making in managing AUS nodules.

## 2. Materials and Methods

### 2.1. Study Design and Patient Selection

This study was conducted across four main stages: patient selection based on inclusion and exclusion criteria, collection of data related to hematologic indices and demographic information, model development using logistic regression analysis, and model validation to assess predictive performance.

From 2018 to 2022, patients who underwent thyroid surgery at the Wonju Severance Christian Hospital (Wonju, Republic of Korea) after being diagnosed with AUS through FNA were retrospectively selected and reviewed. A total of 662 patients fell under this category. The FNA results were classified according to the Bethesda System (2nd version) during the selection process. Among them, 280 patients underwent surgery, and their final histopathological examination results were confirmed. These 280 patients were enrolled as study subjects.

### 2.2. Hematologic Indices and Laboratory Data

The DNI, neutrophil count, lymphocyte count, platelet count, NLR, and PLR were determined using the closest preoperative laboratory data based on the date of surgery.

### 2.3. Pathological and Ultrasonographic Information

For patients where cancer was pathologically confirmed after surgery, the tumor size was measured based on pathological data. For patients without confirmed cancer, the tumor size was measured using preoperative ultrasonography. In this study, the primary tumor size was defined as the largest dimension of the tumor, as is consistent with standard practice in thyroid cancer assessment. The ultrasonographic risk classification of nodules was assessed according to K-TIRADS 2016.

For precise analysis, patients who were pathologically confirmed to have cancer after surgery were assessed using preoperative imaging examinations (ultrasound or CT) to verify whether the nodules initially reported as AUS before surgery could indeed be identified as cancer. If nodules initially reported to be AUS were not confirmed to be cancer, but other nodules in the same patient were reported to be cancer, we classified them as occult cancer. These cases were ultimately included in the benign group.

### 2.4. Data Collection

**Figure 1:**
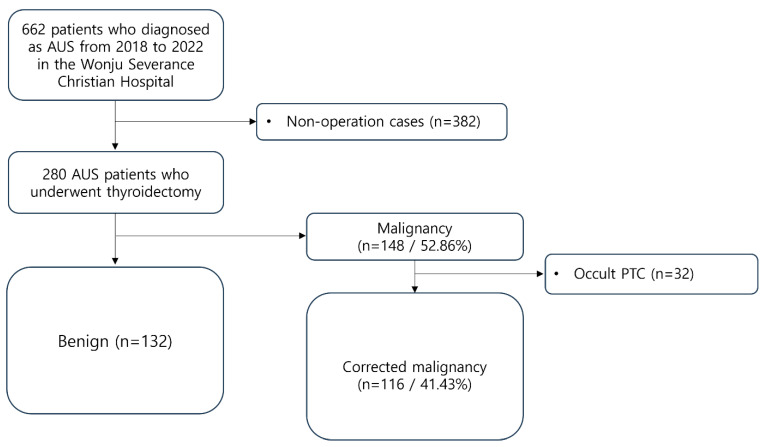
Patient inclusion and exclusion criteria. AUS, atypia of undetermined significance; PTC, papillary thyroid carcinoma.

All data were retrospectively collected based on electronic medical records (EMRs). The radiation exposure history and family history of thyroid cancer were investigated in all patients. The inclusion and exclusion criteria applied in this study are outlined in detail in Figure 1.

### 2.5. Statistical Analysis

Statistical evaluation and machine learning tasks were carried out using the R language. All demographic and clinical variables were compared between the benign and malignancy groups using Student’s *t*-test or Fisher’s exact test. To assess the predictive utility of preoperative patient information, including age, sex, K-TIRADS classification, tumor size, history of radiation exposure, familial history of thyroid cancer, DNI, NLR, and PLR, we conducted both univariate and multivariate logistic regression analyses. Due to the limited representation of patients with a history of radiation exposure and familial history of thyroid cancer in the overall study cohort, these variables were excluded from the multivariate analysis.

For model development and validation, the dataset was divided into training and testing sets using repeated cross-validation (CV). A single loop of 4-fold CV included a partitioning task, whereby the dataset was divided into 4 equal-sized subsets or “folds”; then, for each iteration, 1 fold was used as the testing dataset, and the remaining 3 folds were used as the training dataset. In total, 250 iterations of 4-fold CV generate 1000 random training datasets and their corresponding testing datasets from an original dataset. Logistic regression was employed to train the models on each of these training datasets, resulting in 1000 sets of model coefficients. The performance of each model was then evaluated on the testing datasets using the AUROC as the primary performance metric. AUROC values were calculated for each iteration, and boxplots were used to depict the distribution of these AUROC values across the 1000 iterations.

The final malignancy prediction model was constructed using multivariate logistic regression. Logistic regression (LR) was selected to model the binomial distribution of thyroid malignancy as the dependent variable. LR was chosen for its high explicability, which allows for a clear interpretation of the contribution of each variable to malignancy status. This approach aligns with the study’s objectives of emphasizing variable selection and reliability. As we aimed to predict the binomial distribution of thyroid cancer status, we used multivariate logistic regression including the thyroid cancer condition as dependent variables and candidate predictors as covariates. Model parameters (beta coefficients) were estimated as the mean values of the coefficients obtained from the 1000 training iterations. The AUROC was used to evaluate the model’s classification performance on the testing datasets, with AUROC values averaged across the 1000 iterations to provide a robust measure of model performance. The optimal threshold for the thyroid cancer prediction index was determined by maximizing the F1 score across the 1000 iterations, with the average of these optimal thresholds used as the cut-off for predicting thyroid cancer from AUS.

## 3. Results

The clinicopathological characteristics of the study cohort are given in Table 1. The mean age of all patients was 56.2 years, with 82.9% being female. The primary tumor size averaged 1.86 cm. The K-TIRADS classification revealed 135 cases (48.2%) to be class 4, 94 cases (33.6%) to be class 3, 44 cases (15.7%) to be class 5, and 7 cases (2.5%) to be class 2. The mean values for DNI, NLR, and PLR were 0.17, 2.08, and 148.3, respectively.

Among the entire patient group, 148 individuals (52.86%) were reported as having thyroid cancer. Excluding 32 occult cancer patients, classified based on the aforementioned criteria, a total of 116 patients (41.43%) were classified into the malignancy group (Figure 1). Of the diagnosed cancer cases, 56 (48.3%) were follicular-variant PTC, followed by 47 (40.5%) with classic PTC, 9 (7.8%) with follicular thyroid carcinoma, 3 (2.6%) with Hurthle cell carcinoma, and 1 (0.9%) with medullary thyroid carcinoma (Table 2).

In the univariate analysis, the K-TIRADS classification and tumor size showed statistically significant associations with cancer diagnosis. In the multivariate analysis for the entire patient group, the K-TIRADS classification, tumor size, and age remained significant. When DNI, NLR, and PLR were individually added as variables in the multivariate analysis, none exhibited statistical significance (Table 3).

Considering the widely adopted eighth AJCC/TNM cancer staging system, which divides patients into age groups of <55 years (young patients) and ≥55 years (elderly patients), multivariate analyses were separately conducted. In the elderly patient group, none of the variables demonstrated statistically significant predictive ability. In the young patient group, age, K-TIRADS classification, and tumor size were significant variables, while DNI, NLR, and PLR showed no statistical significance.

**Figure 2:**
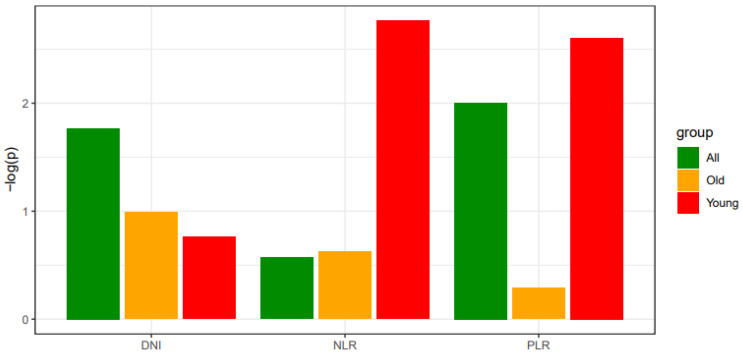
Comparison of statistical significance across study subgroups for laboratory data. The degree of significance (*p*-values) of DNI, NLR, and PLR was evaluated using logistic regression analysis, including the binomial distribution of thyroid cancer and laboratory indices as dependent and independent variables, respectively. The logistic model was applied to the entire patient cohort, the older patient (≥55 years) group, and the younger (<55 years) patient group, separately. The x-axis denotes the subgroup analysis according to different age groups. The y-axis indicates a negative log-transformed *p*-value evaluated using logistic regression. DNI, delta neutrophil index; NLR, neutrophil–lymphocyte ratio; PLR, platelet–lymphocyte ratio.

In the multivariate analysis, the hematologic indices DNI, NLR, and PLR were not independently significant predictors for the entire patient group, the elderly patient group, or the young patient group. However, for NLR and PLR, a trend toward proximity to a *p*-value of 0.05 was observed in the young patient group compared to the entire patient group. To emphasize the statistical significance across age groups, a negative log transformation of *p*-values was applied. This transformation helps to clarify the trend observed in Figure 2.

Based on the statistical analysis, the thyroid cancer prediction model was developed by dividing the patients into two groups: young patients (<55 years old) and older patients (≥55 years old). The group classified using the variables of age, sex, tumor size, and K-TIRADS was designated as the “base” model, while the groups including DNI, NLR, and PLR in addition to the base model were referred to as the “DNI” model, “NLR” model, and “PLR” model, respectively.

**Figure 3:**
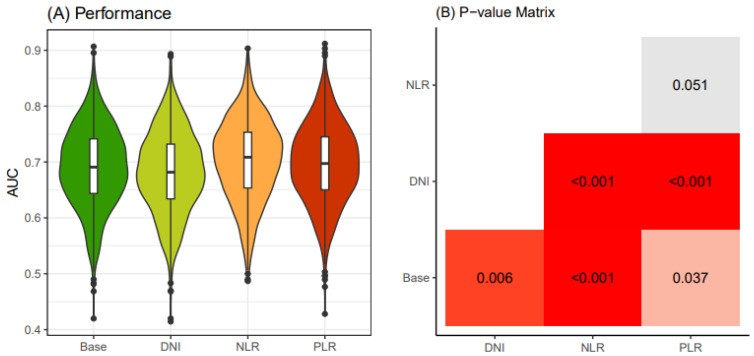
Comparison of performance of four thyroid cancer prediction models (<55 years old). Four classification models were established according to four different combinations of clinical biomarkers. The “base” model includes age, sex, primary tumor size, and K-TIRADS classification as the input variables. The “DNI” model integrates DNI alongside the variables in the base model. In the “NLR” model, NLR is included along with the base model variables. Similarly, the “PLR” model incorporates PLR together with the base model variables. An iteration of four-fold cross-validation was conducted 250 times on an original dataset obtained from the WSCH, yielding 1000 sample training datasets. Logistic regression was implemented to establish the classification model for the binomial distribution of thyroid malignancy status (thyroid cancer vs. benign nodule). The logistic model was iteratively run for the 1000 random training datasets, yielding 1000 performance values. The 1000 performance levels were obtained from the 1000 matched sampling testing datasets. A boxplot depicts the distribution of the 1000 performance measures. In Panel (**A**), different colors are used to visually distinguish between the four models (Base, DNI, NLR, PLR). These colors do not carry any specific quantitative meaning. In Panel (**B**), the colors in the matrix represent the magnitude of *p*-values, with deeper shades of red indicating smaller *p*-values. Gray cells represent *p*-values >0.05, indicating no statistical significance. Exact *p*-values are displayed within each cell for clarity. K-TIRADS, Korean Thyroid Imaging Reporting and Data System; DNI, delta neutrophil index; NLR, neutrophil–lymphocyte ratio; PLR, platelet–lymphocyte ratio; WSCH, Wonju Severance Christian Hospital.

In the young patient group (<55 years old), we computed 1000 AUC values for the four models of preoperative biomarkers after conducting 250 × 4 CVs (cross-validations) on the electronic medical record dataset. Our results indicated that the “NLR” model exhibited superior cancer-predictive performance compared to the other models (Figure 3A). We validated the significantly higher predictive accuracy of the NLR model compared to other models using the Bonferroni-adjusted method (Figure 3B).

**Figure 4:**
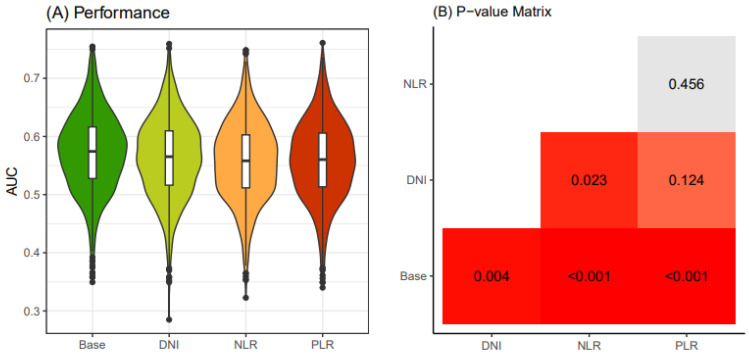
Comparison of performance of four thyroid cancer prediction models (≥55 years old). Four classification models were devised utilizing different combinations of clinical biomarkers. The “base” model was constructed with age, sex, primary tumor size, and K-TIRADS classification as the input variables. The “DNI” model featured DNI in conjunction with the base model variables. The “NLR” model included NLR along with the base model variables, while the “PLR” model incorporated PLR together with the base model variables. An iterative process of four-fold cross-validation was conducted 250 times on an initial dataset sourced from the WSCH, resulting in the generation of 1000 training datasets through sampling. Logistic regression was employed to construct a classification model for the binomial distribution of thyroid malignancy status (thyroid cancer vs. benign nodule). The logistic model underwent iterative executions across the 1000 random training datasets, producing 1000 performance values. These performance levels were derived from 1000 corresponding testing datasets obtained through matched sampling. The distribution of the 1000 performance measures is visually represented in a boxplot. In Panel (**A**), different colors are used to visually distinguish between the four models (Base, DNI, NLR, PLR). These colors do not carry any specific quantitative meaning. In Panel (**B**), the colors in the matrix represent the magnitude of *p*-values, with deeper shades of red indicating smaller *p*-values. Gray cells represent *p*-values >0.05, indicating no statistical significance. Exact *p*-values are displayed within each cell for clarity. K-TIRADS, Korean Thyroid Imaging Reporting and Data System; DNI, delta neutrophil index; NLR, neutrophil–lymphocyte ratio; PLR, platelet–lymphocyte ratio; WSCH, Wonju Severance Christian Hospital.

Similarly, in the older patient group (≥55 years old), following the same procedure, the AUC values of the base model were consistently below 0.6, and the models with additional DNI, NLR, and PLR exhibited even lower AUC values than the base model (Figure 4). Consequently, due to the statistical challenges of establishing prediction models based on these four models, we decided to create prediction models only for the young patient group.

**Figure 5:**
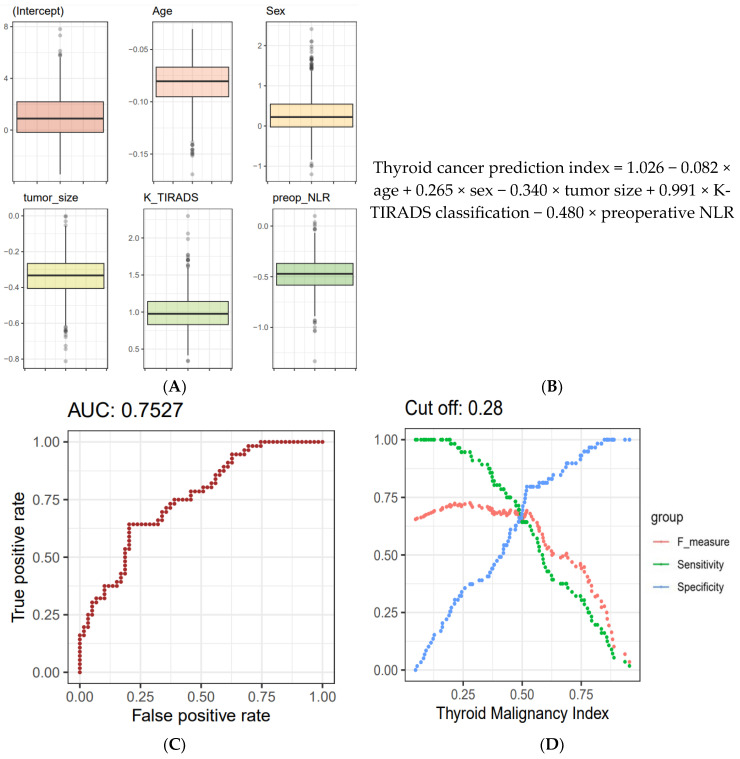
Final malignancy prediction model. (**A**) Iteration of 4-fold cross-validation was applied 250 times to an original dataset obtained from the WSCH, yielding 1000 sample training datasets. Multivariate logistic regression was iteratively used for the 1000 random training sets to generate the binomial distribution of thyroid malignancy status (thyroid cancer vs. benign nodule), yielding 1000 beta coefficients per variable. (**B**) The equation for the thyroid cancer prediction model was established by computing the mean value of the 1000 parameters for each predictor. (**C**) AUROC was used to evaluate the classification performance for the dichotomous status of thyroid cancer condition. AUROC was measured using the 1000 testing datasets matched with the 1000 training sets obtained from the 250 × 4 CV. (**D**) The LR-based thyroid cancer index provides predictive values ranging from 0 to 1. Then, we selected the cut-off of the thyroid cancer index showing the maximum value of the F1 score. We employed 250 × 4 CV for the selection of the optimal predictive value of thyroid cancer, yielding 1000 cut-off values of the thyroid cancer index. The average of the 1000 cut-offs of the LR indices was identified as the optimal threshold for predicting thyroid cancer from AUS. K-TIRADS, Korean Thyroid Imaging Reporting and Data System; NLR, neutrophil–lymphocyte ratio; AUC, area under the curve; WSCH, Wonju Severance Christian Hospital; AUROC, area under the ROC curve; LR, logistic regression; CV, cross-validation; AUS, atypia of undetermined significance.

The beta coefficients for each biomarker were derived using logistic regression (LR) analysis. To ensure generalizability, a 250 × 4 CV approach was used to generate 1000 training sets; applying LR to these sets resulted in 1000 lists of coefficients following a Gaussian distribution, in line with the central limit theorem (Figure 5A). The average value from these distributions was adopted as the final parameter for the thyroid cancer prediction equation (Figure 5B). To determine the cut-off value for the thyroid cancer prediction index from the LR-based network, a four-fold cross-validation was iterated 250 times. This process produced 1000 distributions of F-scores, from which the maximum F-score values were extracted. Ultimately, the optimal cut-off value of 0.28 for the thyroid cancer prediction index was established (Figure 5D).

## 4. Discussion

The current study was designed to identify factors aiding in the prediction of cancer in patients diagnosed with AUS. This research sought to address the incomplete nature of the treatment guidelines for AUS, which, unlike Bethesda categories IV (follicular neoplasm) and V (suspicious for malignancy), suggests repeat FNA and molecular testing as the basic principles for AUS diagnosis [1,8,9].

However, many reports indicate that repeating FNA in AUS often results in the same diagnosis being given again. In Korean guideline publications, it is noted that repeat FNA examinations may exhibit results that are over 60% inconclusive. The guidelines express some skepticism regarding repeat FNA and suggest that, to overcome this issue, performing a core needle biopsy during reassessment for AUS could be considered an alternative option for reducing the rate of inconclusive results [8].

In the realm of molecular biological testing, availability is currently limited to BRAF mutations in PTC in real-world scenarios, necessitating additional research for other gene mutations. The BRAF V600E mutation is effectively the sole molecular marker used to predict thyroid cancer in clinical settings. In a meta-analysis of 18 studies, out of 2766 thyroid FNA samples, 581 were positive for the BRAF mutation, among which 580 were ultimately diagnosed with PTC. Even when considering one sample that was not diagnosed with PTC but showed a benign nature as a false negative, the rate of malignancy in BRAF-positive nodules was 99.8% [10]. Furthermore, in other studies, when retrospectively examining BRAF-positive FNA samples, 15–39% of the samples were reported to have indeterminate or non-diagnostic results in cytology. This strongly suggests that BRAF testing could be a valuable tool in diagnosing cancer in cases of indeterminate cytology [11].

Another gene mutation test that can be considered for use in PTC is the TERT mutation test. This test is particularly applicable to patients who are older and have a higher likelihood of lymph node metastasis and distant metastasis, as well as a higher probability of showing resistance to radioactive iodine therapy. When accompanied by a BRAF mutation or RAS mutation, it may indicate a worse prognosis and can be used as a prognostic factor [12]. However, this mutation exhibits high specificity but low sensitivity. A recent prospective meta-analysis involving 3366 PTC patients in South Korea revealed that only 2.6% showed this mutation. This suggests that its practical utility as a predictive factor in clinical settings may be somewhat limited [13]. Therefore, this genetic mutation is more commonly used as a prognostic marker than a diagnostic marker.

RAS mutations are most commonly observed in follicular thyroid carcinoma (FTC), appearing in 40–50% of cases of this cancer subtype. However, this mutation can also be detected in benign nodules and non-invasive follicular thyroid neoplasms with papillary-like nuclear features (NIFTPs), contributing to an increased false positive rate for RAS mutation detection. For example, in a meta-analysis focusing on cases categorized as category III and IV, RAS mutations were reported to have a positive predictive value (PPV) of 66% for thyroid malignancy [14]. In another study focusing on Bethesda categories III, IV, and V, RAS mutations were reported to have a risk of malignancy (ROM) of 76% for thyroid cancer [15].

Research is being conducted into NGS-based multi-gene analysis technology, which uses a multi-gene assay based on NGS to predict malignancy, encompassing representative gene mutations such as RET, PAX8, and PPAR, among others, as previously discussed. Among these, ThyroSeq v3, a well-known assay kit, was evaluated in a study targeting 175 patients with Bethesda categories III, IV, and V. The kit demonstrated a sensitivity of 98%, specificity of 82%, and accuracy of 91% in distinguishing thyroid malignancy [16]. This study encompassed all types of thyroid malignancies, including DTC, PDTC, ATC, and MTC. However, the high costs (exceeding USD 3000) associated with this technology remain a significant drawback, and its practical implementation and commercialization still require further time and development.

In addition to gene mutation, immunohistochemistry is being explored as a supplementary method for determining the malignancy of indeterminate nodules. Key studies include those on galectin-3, HBME-1, CK19, and the loss of CD 56 expression. In a multicenter study using 465 FNA samples diagnosed as follicular neoplasm pre-surgery, galectin-3 positivity was observed in 134 of the 465 samples, of which 101 (75%) were cancerous. It was also found that 29 galectin-3-negative samples were reported as cancerous. The study concluded that the galectin-3 test had a sensitivity of 78%, a specificity of 93%, and a positive predictive value of 82% [17].

However, this value is deemed to be too low for use as a standalone predictor of malignancy in actual clinical practice, and it is evident that several studies on the aforementioned immunohistochemistry markers, aside from galectin, also demonstrate relatively low sensitivity and positive predictive values. To address this shortcoming, recent efforts have focused on employing a combination of various immunohistochemical markers. Notably, the amalgamation of galectin-3, CK19, and HBME1 has been documented in multiple studies. In research involving 66 patients with follicular adenoma (FA) and 66 patients with PTC, this trio of staining techniques yielded a PPV of 97% and a negative predictive value (NPV) of 96% in diagnosing PTC. Nonetheless, this study has its limitations, including the absence of an analysis of PTC variants and the lack of confirmation regarding the inclusion of the follicular variant of papillary thyroid carcinoma (FVPTC) [18]. Similarly, another study that utilized the combination of these three staining methods analyzed 231 patients and reported a PPV of over 97% for thyroid cancer. However, this study also had limitations because it was conducted using only the binary classification of benign and malignant, regardless of the specific type of cancer [19]. In another study involving 27 patients with follicular adenoma (FA) and 45 patients with the FVPTC, it was reported that the combination of these three staining methods showed a sensitivity of 87% and a specificity of 89% in diagnosing the FVPTC [20].

As such, despite the guidelines recommending repeat FNA and molecular biological testing, there are still vulnerabilities and areas that require further research. An additional consideration for the treatment of AUS is that, compared to the reported ROM, a higher actual ROM is reported in various studies [21,22,23]. The proportion of patients reported as cancerous in this study, excluding occult cancer patients, also surpassed the 13–30% risk of malignancy reported in the existing literature, suggesting potential limitations in the current somewhat conservative approach compared to surgical treatment. Therefore, the development of a new tool to determine whether AUS ultimately leads to a cancer diagnosis is crucial for deciding on an appropriate surgical treatment policy.

We aimed to address this perspective by creating a malignancy prediction model that combines hematologic indices such as DNI, NLR, and PLR with well-known preoperative risk factors for thyroid cancer. The statistical potential to create such a model was more evident in the young patient group than in the entire patient group, based on a multivariate analysis. Upon confirming the statistical trends in P-values, we decided that combining NLR with age, sex, tumor size, and K-TIRADS classification in a feature set could enhance predictive power. Through the integration of a range of variables, a prediction model was developed to forecast thyroid cancer in cases of AUS, utilizing logistic regression via machine learning techniques. The optimal cut-off value was determined using the F1 score.

Contrary to our initial expectations and previous findings, our prediction model revealed a negative correlation between NLR and the likelihood of thyroid cancer, with higher NLR values associated with a decreased malignancy risk. The reason for this unexpected outcome is not fully understood and may reflect specific characteristics within our cohort. Furthermore, the relatively small sample size of younger patients suggests a need for larger, multicenter studies to clarify the relationship between NLR and thyroid cancer risk, as well as to confirm whether this trend persists across broader populations. Additionally, this study includes patients previously or subsequently diagnosed with thyroid conditions such as Graves’ disease and Hashimoto’s thyroiditis, both of which are associated with chronic inflammatory responses. These autoimmune conditions can lead to elevated NLR values, as evidenced by studies linking higher NLR to increased relapse risk in Graves’ disease and elevated baseline levels in Hashimoto’s thyroiditis [24,25]. This inflammatory effect may have influenced NLR and PLR distribution in our cohort, potentially confounding their predictive accuracy for malignancy and representing a limitation of our study.

Furthermore, preoperative factors that proved statistically useful in predicting cancer among young patients were found to be insignificantly predictive in older patients, a trend also observed in hematological indices including the DNI, NLR, and PLR. Internationally recognized thyroid cancer classification systems, including the eighth edition of the American Joint Committee on Cancer (AJCC), set the age threshold at 55 years, indicating that cancers tend to be more aggressive and the prognoses worse in individuals older than 55. This adjustment was incorporated in the seventh edition based on empirical studies [26]. Therefore, it can be inferred that thyroid cancer in individuals over 55 years of age will have a pathophysiology distinct from that in younger patients. This difference may explain why factors significant in the young patient group become much less meaningful in the older patient group.

Interestingly, younger patients (under 55 years old) have been shown to have a higher likelihood of AUS nodules being diagnosed as malignant compared to older patients [27]. This result underscores the unique characteristics of younger patients with AUS nodules, suggesting that factors such as immune system activity, hormonal differences, or genetic predispositions may influence the malignancy risk in this population. In our study, a further analysis within the under-55 subgroup revealed that younger age was also a significant predictor of malignancy, with the likelihood of malignancy decreasing incrementally as age increased (OR = 0.928, *p* = 0.012). These findings highlight the need for tailored approaches to risk stratification in AUS nodules, both between different age groups and within the younger population. Additionally, elderly patients are more likely to have concomitant underlying diseases compared to younger patients. Indices such as DNI, NLR, and PLR are associated not only with thyroid cancer but also with many other types of carcinomas, infectious diseases, and respiratory–circulatory diseases, which could have affected the statistical analysis. Additionally, in elderly patients, as aging progresses, chronic inflammatory status becomes widespread, and both cell regeneration capacity and phagocytosis decrease. Hence, it is plausible to hypothesize that the sensitivity and predictive accuracy of indicators including DNI, NLR, and PLR would be lower in the older patient group compared to the younger one [28,29,30]. In attempts to improve predictive accuracy for the older patient group, we explored alternative model adjustments, but these modifications did not yield statistically significant improvements. Therefore, our final model focused on the younger group, where the predictive power of the DNI, NLR, and PLR markers was more robust.

To further explore the role of NLR in thyroid cancer, we examined its biological relevance in the context of systemic inflammation and immune modulation. The neutrophil-to-lymphocyte ratio (NLR) reflects the systemic inflammatory response and immune status of the host, which are critical in the development and progression of malignancies. Elevated NLR is indicative of neutrophil-driven pro-tumor inflammation and a concurrent suppression of lymphocyte-mediated anti-tumor immunity. This imbalance is particularly relevant in thyroid cancer, where inflammation and immune modulation play a central role in tumor progression [31,32].

In thyroid cancer, neutrophils contribute to tumorigenesis by producing cytokines and growth factors such as vascular endothelial growth factor (VEGF), which promote angiogenesis and tumor invasiveness. At the same time, lymphocytes—particularly cytotoxic T cells—are critical for tumor surveillance and eradication, and their reduction further compromises anti-tumor immunity [33]. These processes are reflected in elevated NLR levels, which have been associated with aggressive features of thyroid cancer, including extrathyroidal extension, lymph node metastasis, and larger tumor size [31,33].

The biological relevance of NLR in thyroid cancer is supported by its ability to capture systemic inflammatory states that are linked to tumor behavior. For example, in radioiodine-refractory differentiated thyroid cancer, elevated NLR has been independently associated with worse survival outcomes, emphasizing its prognostic significance in advanced disease [33]. These findings suggest that NLR not only serves as a predictive biomarker but also provides insights into the underlying pathophysiology of thyroid cancer.

In our study, the inclusion of NLR as a variable significantly improved malignancy prediction accuracy in AUS nodules. This highlights the potential of inflammatory markers such as NLR to enhance preoperative risk stratification and guide clinical decision making in the management of thyroid cancer.

This study has several limitations that should be acknowledged. First, the retrospective nature of the study may introduce inherent biases, such as incomplete datasets or variability in the quality of medical records, which could influence the results. Second, the study population was derived from a single institution, potentially limiting the generalizability of the findings for broader populations or diverse clinical settings. This selection bias may also result in an overrepresentation or underrepresentation of specific subgroups.

Additionally, the relatively small sample size, particularly in the younger patient subgroup, may limit the statistical power of the analysis. This could impact the reliability of the observed associations between variables such as NLR and the malignancy risk. Furthermore, the inclusion of patients with coexisting autoimmune thyroid diseases, such as Graves’ disease and Hashimoto’s thyroiditis, may have confounded the hematologic indices, including NLR and PLR, as these conditions are associated with systemic inflammation, which could influence their predictive accuracy for malignancy.

Finally, this study primarily focused on preoperative risk factors and did not incorporate postoperative outcomes, which could provide additional insights into the clinical implications of the findings. Future studies should aim to address these limitations by including larger, multicenter cohorts and incorporating prospective designs to validate the results.

## 5. Conclusions

The development of this new prediction model has the potential to aid in determining suitable treatment policies for younger patients with atypia of undetermined significance (AUS). A notable advantage of this predictive model is that it incorporates easily accessible indicators in terms of cost-effectiveness for most hospital facilities, as opposed to gene mutation testing or immunohistochemistry testing, which generally entail relatively high costs.

Clinically, this model can guide more accurate preoperative risk stratification by prioritizing high-risk patients for early interventions, while reducing unnecessary repeat biopsies or surgeries for low-risk cases. Such an approach may help to improve patient outcomes by minimizing overtreatment and enhancing the efficiency of healthcare delivery. While this study provides promising insights, the limited cohort size and single-center design may impact the model’s generalizability. A larger, multicenter cohort is necessary to assess and enhance the model’s robustness, allowing for broader applicability across diverse patient populations before clinical implementation.

## Figures and Tables

**Table 1:** Preoperative baseline characteristics.

Characteristics	All ^a^	Benign ^a^	Malignancy ^a^	*p*
	(n = 280)	(n = 164)	(n = 116)	
Age, years, mean ± SD	56.2 ± 12.1	57.3 ± 10.4	54.5 ± 14.0	0.061
Sex				0.721
Female	232 (82.9)	27 (16.5)	21 (18.1)	
Male	48 (17.1)	137 (83.5)	95 (81.9)	
Primary tumor size, cm	1.86 ± 1.45	2.05 ± 1.65	1.59 ± 1.06	0.005
K-TIRADS				0.001
2	7 (2.5)	5 (3)	2 (1.7)	
3	94 (33.6)	65 (39.6)	29 (25)	
4	135 (48.2)	77 (47)	58 (50)	
5	44 (15.7)	17 (10.4)	27 (23.3)	
History of radiation exposure, n	4 (1.4)	2 (1.2)	2 (1.7)	0.727
Familial history of thyroid cancer, n	12 (4.3)	7 (4.3%)	5 (4.3)	0.986
DNI, %	0.17 ± 0.60	0.23 ± 0.73	0.1 ± 0.32	0.047
NLR	2.08 ± 1.01	2.06 ± 1.03	2.04 ± 0.97	0.868
PLR	148.3 ± 59.96	152.26 ± 69.85	142.7 ± 41.88	0.155

^a^ Data are expressed as *n* (%) unless otherwise noted. SD, standard deviation; K-TIRADS, Korean Thyroid Imaging Reporting and Data System; DNI, delta neutrophil index; NLR, neutrophil–lymphocyte ratio; PLR, platelet–lymphocyte ratio.

**Table 2:** Pathologic information about thyroid cancer patients.

	Malignancy (n = 116) ^a^
Cancer type	
Classic papillary thyroid carcinoma	47 (40.5)
Follicular-variant papillary thyroid carcinoma	56 (48.3)
Follicular thyroid carcinoma	9 (7.8)
Hurthle cell carcinoma	3 (2.6)
Medullary thyroid carcinoma	1 (0.9)
Extrathyroidal extension	
No	99 (80.2)
Minimal	17 (14.7)
Multifocality	42 (36.2)
Bilaterality	23 (19.8)
Lymphatic invasion	4 (3.4)
Vascular invasion	0
Perineural invasion	3 (2.6)
Central lymph node metastasis	11 (9.5)

^a^ Data expressed as *n* (%) unless otherwise noted.

**Table 3:** Association of preoperative variables with the diagnosis of malignancy in patients with atypia of undetermined significance nodules.

Characteristics	Univariate		Multivariate					
			All		Older Patients (≥ 55 Years Old)		Young Patients (<55 Years Old)	
	OR (95% CI)	*p*	OR (95% CI)	*p*	OR (95% CI)	*p*	OR (95% CI)	*p*
Age, years	0.98 (0.961–1)	0.051	0.979 (0.959–1)	0.048	1.05 (0.999–1.104)	0.057	0.928 (0.876–0.984)	0.012
Female	0.892 (0.476–1.67)	0.72	0.764 (0.396–1.474)	0.422	0.588 (0.255–1.357)	0.213	1.013 (0.32–3.204)	0.982
K-TIRADS classification	1.783 (1.268–2.506)	0.001	1.718 (1.208– 2.443)	0.003	1.353 (0.865–2.116)	0.185	2.588 (1.369–4.894)	0.003
Primary tumor size, cm	0.782 (0.648–0.944)	0.01	0.812 (0.671–0.983)	0.033	0.792 (0.601–1.042)	0.096	0.739 (0.548–0.997)	0.048
History of radiation exposure *	1.421 (0.197–10.237)	0.727		-	-			
Familial history of thyroid cancer *	1.01 (0.313–3.265)	0.986		-	-			
DNI **	0.639 (0.378–1.079)	0.094	0.689 (0.404–1.175)	0.171	0.727 (0.362–1.463)	0.372	0.725 (0.304–1.727)	0.467
NLR **	0.98 (0.772–1.243)	0.867	0.928 (0.721–1.195)	0.564	1.108 (0.802–1.532)	0.534	0.627 (0.383–1.026)	0.063
PLR **	0.997 (0.993– 1.001)	0.194	0.996 (0.991–1.001)	0.135	0.999 (0.993–1.005)	0.749	0.993 (0.984–1.001))	0.074

Univariate logistic regression was used to determine the association between candidate markers (independent variable) and the binomial malignant status (yes or no, dependent variable). Multivariate analysis was conducted with age, sex, K-TIRADS classification, and primary tumor size as independent or confounding variables. * Very few subsets of enrolled patients had a history of radiation exposure and a family history of thyroid cancer, leading to their exclusion from the multivariate analysis. ** Additional analyses were performed by including DNI, NLR, and PLR as separate independent variables. The table presents the resulting odds ratio (OR) and *p*-values for each variable. OR, odds ratio; CI, confidence interval; K-TIRADS, Korean Thyroid Imaging Reporting and Data System; DNI, delta neutrophil index; NLR, neutrophil–lymphocyte ratio; PLR, platelet–lymphocyte ratio.

## Data Availability

The data presented in this study are available upon request from the corresponding author. The data are not publicly available due to the privacy of the enrolled patients.
